# Tetracycline Resistance Among Canine Methicillin-Resistant *Staphylococcus pseudintermedius* (MRSP) Clinical Isolates: Is Minocycline a Viable Treatment Option?

**DOI:** 10.3390/antibiotics15010009

**Published:** 2025-12-20

**Authors:** Magdalena Kizerwetter-Świda, Dorota Chrobak-Chmiel, Ilona Stefańska, Ewelina Kwiecień, Rafał Nejfeld, Magdalena Rzewuska

**Affiliations:** Department of Preclinical Sciences, Institute of Veterinary Medicine, Warsaw University of Life Sciences, Ciszewskiego 8, 02-786 Warsaw, Poland; dorota_chrobak@sggw.edu.pl (D.C.-C.); ewelina_kwiecien1@sggw.edu.pl (E.K.); rafal_nejfeld@sggw.edu.pl (R.N.); magdalena_rzewuska@sggw.edu.pl (M.R.)

**Keywords:** methicillin-resistant *Staphylococcus pseudintermedius*, tetracyclines resistance, minocycline, MLST

## Abstract

**Background/Objectives**: Infections caused by multidrug-resistant methicillin-resistant *Staphylococcus pseudintermedius* (MRSP) strains are becoming increasingly common in veterinary medicine. Depending on which *tet* genes are present, MRSP isolates may exhibit resistance to all tetracyclines or resistance to tetracycline and doxycycline with susceptibility to minocycline. As minocycline may be a potential treatment option, our study aimed to verify this hypothesis. We have compared phenotypic resistance to tetracyclines with the presence of the *tet*(K), *tet*(L), *tet*(M), and *tet*(O) genes and conducted the molecular characterization of 50 clinical MRSP isolates of canine origin. **Methods**: The presence of the *tet* genes was determined by PCR. Molecular characterization included multilocus sequence typing (MLST) and staphylococcal cassette chromosome *mec* (SCC*mec*) typing. **Results**: Among the 50 examined clinical MRSP isolates, sequence type ST71 with the SCC*mec* II–III cassette was predominant (n = 27; 54%). Among these isolates, the *tet* genes were either absent or present only as the tet(K) gene, resulting in susceptibility to all tetracyclines, tetracycline and doxycycline resistance, and minocycline susceptibility. In contrast, isolates carrying the SCC*mec* type V cassette (n = 20; 40%) were resistant to all tetracyclines tested and belonged to ST551, ST2851 (new), ST2869 (new) and ST672. These genotypes were most often characterized by the presence of a single *tet*(M) gene; two genes, *tet*(M) and *tet*(K); or three genes, *tet*(M), *tet*(K) and *tet*(O). Notably, 28 out of 50 isolates (56%) showed minocycline susceptibility, and 19 (38%) were minocycline-susceptible and resistant to tetracycline and doxycycline. **Conclusions**: The obtained results indicate that genotype ST551 and its related ST2851 carry the SCC*mec* type V and typically contain two or even three *tet* genes with the *tet*(M) gene, which confers resistance to all tetracyclines, including minocycline. These genotypes are becoming more common in Poland, and thus, minocycline may be unsuitable for the treatment of MRSP infections in our geographical region. However, in other countries, distinct MRSP genotypes exhibiting minocycline susceptibility may predominate, such as those belonging to clonal complex 71 and carrying the SCC*mec* type II–III cassette. In the case of such strains, minocycline may be a therapeutic option. Therefore, it is advisable to monitor the spread of MRSP clones carrying different *tet* genes and exhibiting varying minocycline resistance profiles.

## 1. Introduction

Methicillin-resistant *Staphylococcus pseudintermedius* (MRSP) is a multidrug-resistant, opportunistic pathogen that primarily affects dogs and is rarely found in other animal species [1,2]. Occasionally, it may also cause zoonotic infections, particularly among persons in close contact with dogs, such as dog owners and veterinarians [3,4]. The spread of zoonotic staphylococci is facilitated by close in-house contact, such as sharing a bed with dogs or being licked by them. However, the transfer of resistance genes may also occur among various bacteria, as confirmed by the literature [5].

The number of antimicrobial agents potentially active against multidrug-resistant staphylococci is quite limited [1,6,7,8]. Resistance to tetracyclines among MRSP strains is relatively common, with reported prevalence ranging from approximately 20% (16.3% in Sweden; 21.7% in Argentina) to over 70% (74% in Finland; 85.7% in South Africa), depending on geographic region and study population [8,9,10,11,12]. However, depending on the molecular mechanism, resistance to all tetracycline drugs may be observed, or strains may be resistant to antimicrobials of this group, except for minocycline, which remains a potential therapeutic option for these bacteria [13,14].

There are two known mechanisms of tetracycline resistance in staphylococci: ribosomal protection and efflux pumps [15,16]. Protective ribosome modifications in staphylococci are encoded by genes *tet*(M), *tet*(O), *tet*(S), *tet*(W), *tet*(44), and membrane-associated efflux proteins by the genes *tet*(K), *tet*(L), *tet*(38), *tet*(42), *tet*(43), *tet*(45) and *tet*(65) (https://faculty.washington.edu/marilynr/, accessed on 28 November 2025). According to the literature data, *tet*(M), *tet*(K) and *tet*(L) genes are the most prevalent in MRSP isolates, as in other Staphylococci [9,14,17]. The presence of different *tet* genes does not necessarily affect susceptibility to minocycline and glycylcyclines (glycylcyclines are not approved for use in veterinary medicine) [17]. MRSP isolates carrying the *tet*(K) or *tet*(L) genes exhibit resistance to tetracycline and doxycycline, but are susceptible to minocycline. While isolates with the *tet*(M) or the *tet*(O) genes are resistant to all antibiotics from the tetracycline group, including minocycline. Therefore, minocycline may be considered a potential treatment option for MRSP infections [13]. Nevertheless, data on the phenotypic susceptibility of MRSP isolates to minocycline and on the prevalence of genes from the *tet* group, especially *tet*(L) and *tet*(O), are still limited.

Multilocus sequence typing (MLST) of methicillin-resistant *Staphylococcus pseudintermedius* isolates is a molecular technique used in epidemiology, enabling the determination of dominant clones within a population [2,18,19]. In genotypic characterization, staphylococcal cassette chromosome *mec* (SCC*mec*) typing is also often performed [20,21]. Initially, the global MRSP population was dominated by sequence type 71 (ST71), which carried cassette SCC*mec* II–III, and ST68, which carried cassette SCC*mec* V, in Europe and North America, respectively [2,19,20]. Isolates from Asia were more heterogeneous, with the most prevalent being ST45 and ST121. Since 2010, an increase in the diversity of STs has been observed. New clones, such as ST258 and ST45, have emerged in Europe, while ST84, ST150, and ST155 have appeared in North America [21,22,23,24]. In Poland, ST71 dominated until 2015, while in later years ST551 with cassette SCC*mec* V was most frequently detected [25]. These data clearly indicate that the MRSP population is constantly evolving.

Notably, individual MRSP clones are characterized by divergent antimicrobial susceptibility profiles, which result from the presence of different mobile genetic elements that carry distinct resistance genes. By combining MLST with antimicrobial susceptibility testing, it is possible to correlate specific STs with characteristic resistance profiles, track the dissemination of high-risk clones, and monitor the emergence of new lineages [1,2]. This approach offers valuable insights into the evolution of multidrug resistance in MRSP, supporting evidence-based decisions for therapy, infection control, and surveillance in veterinary medicine. As already mentioned, there is a gap in the literature data concerning minocycline resistance and the prevalence of different *tet* genes in specific STs among MRSP isolates.

The objective of this study was to compare phenotypic resistance to tetracycline-class antimicrobials, as determined by the disk diffusion method and minimum inhibitory concentration (MIC) values, with the presence of the *tet*(K), *tet*(L), *tet*(M), and *tet*(O) resistance genes among clinical MRSP isolates of canine origin. Additionally, MLST and SCC*mec* typing were performed to assess minocycline susceptibility among the genotypes identified in the tested MRSP isolates.

## 2. Results

### 2.1. Bacterial Isolates

Bacterial canine isolates were obtained from clinical materials obtained from skin and soft tissue infections (n = 29), urinary tract infections (n = 12) and from other clinical materials, i.e., internal organs (n = 2), nasal swab (n = 2), conjunctiva swab (n = 2), throat swab (n = 1), oral swab (n = 1) and stool sample (n = 1) (Table S1). All isolates showed typical *S. pseudintermedius* colony characteristics and were catalase-positive. After Gram staining, all demonstrated Gram-positive cocci morphology. For all 50 isolates tested, a 926 bp thermonuclease *nuc* gene fragment specific for *S. pseudintermedius* was obtained. Methicillin resistance was confirmed by the oxacillin disk method and by detecting a 532 bp *mecA* gene fragment.

### 2.2. Tetracycline Susceptibility Testing

**Figure 1:**
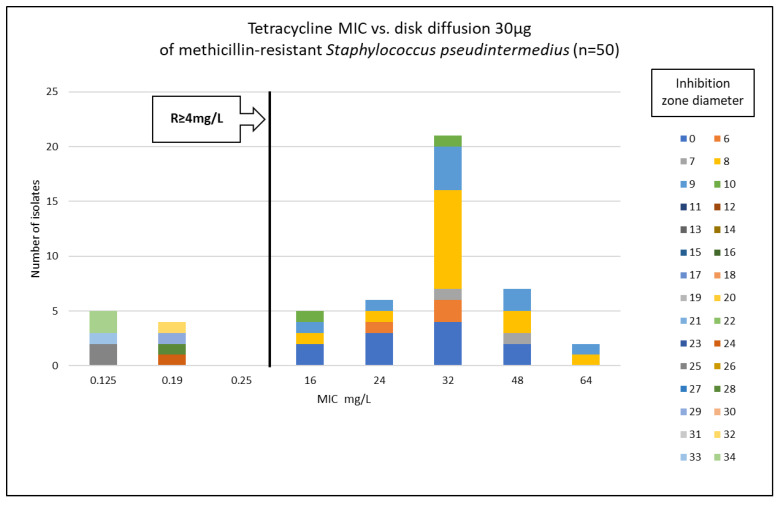
Correlation between tetracycline MIC values and inhibition zone diameters of methicillin-resistant *Staphylococcus pseudintermedius* (n = 50).

**Figure 2:**
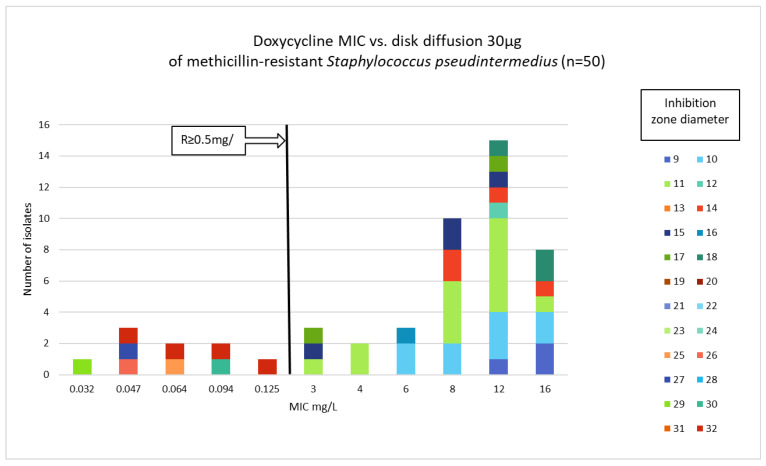
Correlation between doxycycline MIC values and inhibition zone diameters of methicillin-resistant *Staphylococcus pseudintermedius* (n = 50).

**Figure 3:**
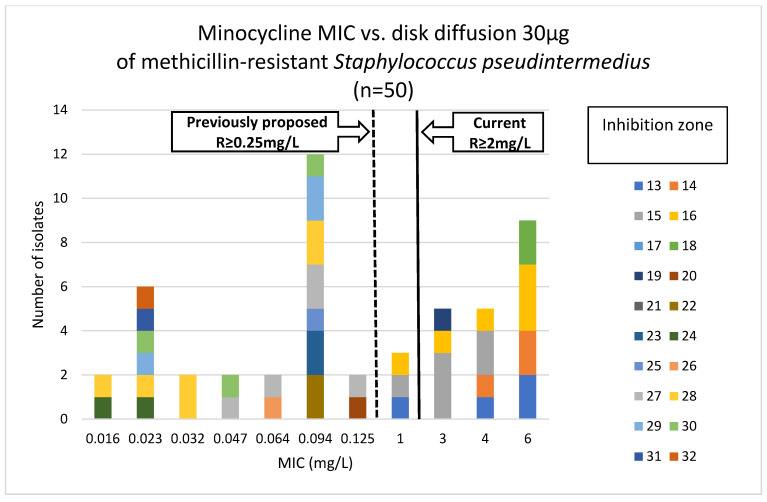
Correlation between minocycline MIC values and inhibition zone diameters of methicillin-resistant *Staphylococcus pseudintermedius* (n = 50).

Phenotypic susceptibility testing results for individual isolates are presented in Table S1. The correlations between MIC values and inhibition zone diameters of tetracycline, doxycycline and minocycline are shown in Figure 1, Figure 2 and Figure 3. The results obtained with the disk diffusion method and MIC values determination were consistent. In general, 9 (18%) out of 50 MRSP isolates were recognized as susceptible to all antimicrobials from tetracycline group. The remaining 41 isolates (82%) exhibited resistance to tetracycline and doxycycline. Among these 41 isolates, 22 were also found to be minocycline-resistant, resulting in 44% of tested MRSP resistant to three tetracyclines used in this study. Additionally, Table 1 presents summary results regarding MIC values, i.e., MIC range, MIC_50_, MIC_90_, and percentage of resistant isolates with confidence intervals (CI = 95%).

### 2.3. Detection of Tetracycline Resistance Genes

Among the tested isolates, the *tet*(K) gene was most frequently detected, either as a single *tet*(K) gene (n = 19; 38%), the *tet*(K) with the *tet*(M) gene (n = 10; 20%), or in a combination of three genes: *tet*(K), *tet*(M) and *tet*(O) (n = 8; 16%). Two genes, *tet*(M) and *tet*(O), were detected in two MRSP isolates. Similarly, two genes, *tet*(M) with *tet*(L), were identified in a single isolate (n = 1; 2%). Additionally, a single *tet*(M) gene was present in one isolate (n = 1; 2%). The tested genes from the *tet* group were not detected in 9 (18%) of the studied isolates, which were also phenotypically susceptible to tetracyclines.

### 2.4. Molecular Characterization of MRSP Isolates

MLST analysis revealed that ST71 was the predominant sequence type among the isolates in this study (n = 29; 58%). The next most frequently detected STs were ST551 (n = 9; 18%) and ST2851 (n = 9; 18%). Additionally, single isolates from other STs were recognized: ST258, ST672 and ST2869. Two novel sequence types, ST2851 and ST2869, were found and deposited in the *S. pseudintermedius* MLST database (https://pubmlst.org/organisms/staphylococcus-pseudintermedius, accessed on 28 December 2025). The results of SCC*mec* typing showed that SCC*mec* II–III was the most prevalent (n = 27; 54%), followed by SCCmec V (n = 20; 40%). The third cassette type detected in the three isolates (6%) tested was SCC*mec* IV.

**Figure 4:**
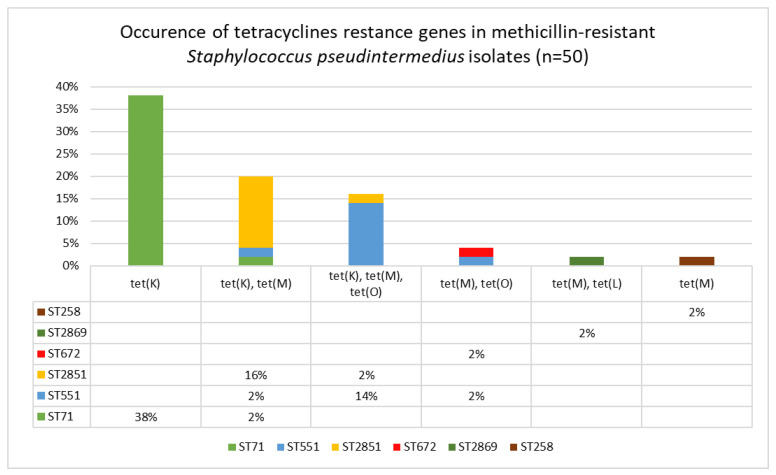
Distribution of tetracycline resistance genes among sequence types of clinical methicillin-resistant *Staphylococcus pseudintermedius* isolates (n = 50).

Comparison of the MLST and the SCC*mec* typing results showed that among the isolates belonging to ST71, the SCC*mec* II–III cassette was the most common. Additionally, these isolates were characterized either by the absence of the *tet* genes or by the presence of a single *tet*(K) gene. The SCC*mec* V cassette was detected only in isolates from ST551, ST2851, and ST672, which also carried either the *tet*(M) gene alone or both the *tet*(M) and *tet*(K) genes. The correlations between detected *tet* genes, STs and SCC*mec* typing results are shown in Figure 4 and Table 2.

Considering the changes in *S. pseudintermedius* over the years of the study (2007–2024), initially, the population of MSRP isolates was dominated by ST71-II–III. However, after 2015, ST551 and new STs were most frequently detected, among which ST2851 predominated.

## 3. Discussion

In this study, we presented the phenotypic susceptibility testing results of 50 canine clinical MRSP isolates to tetracycline group antimicrobials, molecular tetracycline resistance mechanisms, and molecular typing of the isolates. Our results confirmed diverse tetracycline resistance in MRSP isolates from different STs, which carried different SCC*mec* cassettes. Tetracycline resistance was detected in the majority of our isolates (n = 41; 82%). Further, 22 (44%) of these 41 isolates were also resistant to minocycline, while 19 (38%) were susceptible to this antimicrobial but resistant to tetracycline and doxycycline. However, considering all isolates tested, 28 (56%) were found to be susceptible to minocycline. Our results are consistent with literature data indicating a correlation between individual STs and resistance profiles, with minocycline susceptibility occurring significantly more frequently for isolates belonging to ST71 carrying the II–III SCC*mec* cassette.

It should be emphasized that, as mentioned in the introduction, there is a literature gap in data concerning minocycline resistance in methicillin-resistant *S. pseudintermedius*. Most studies report tetracycline or doxycycline resistance testing results, as well as the occurrence of *tet*(K) and *tet*(M) genes [14,24]. Minocycline resistance is less frequently determined. Moreover, even if such data are available, MLST and SCC*mec* typing are often missing. Some studies concern only methicillin-susceptible *Staphylococcus pseudintermedius* (MSSP), and it is known that they have different resistance profiles than MRSP. For this reason, the scarce literature data should be interpreted with caution. Furthermore, MIC breakpoints for minocycline and staphylococci of canine origin were not available until 2024, when CLSI recommendations defined the breakpoint for susceptible isolates as less than or equal to 0.5 mg/L, for intermediate as 1.0 mg/L, and for resistant isolates as greater than or equal to 2.0 mg/L [26]. Results from previous years, when these breakpoints were not accessible, showed that the human tetracycline breakpoints overestimated susceptibility to minocycline for veterinary *S. pseudintermedius* isolates. Moreover, the minocycline breakpoint of 0.25 mg/L or less, proposed by Hnot et al. (2015) [27], was lower than the human breakpoints, but it allowed for obtaining reliable resistance testing results for minocycline and veterinary isolates. Minocycline-resistant MRSP isolates with confirmed presence of *tet*(M) genes were correctly identified using a proposed MIC susceptibility breakpoint [27]. Additionally, the results obtained by Maaland et al. 2014 confirmed the effectiveness of the proposed breakpoint value in relation to PK/PD minocycline data in dogs [13]. Accordingly, it may be assumed that the interpretation of MIC results prior to the release of the current CLSI recommendations differed. Under earlier provisional breakpoints, isolates with MIC values of 0.5 mg/L and 1 mg/L would have been classified as resistant, whereas according to the current recommendations, they are categorized as susceptible and intermediate, respectively.

Notably, three out of 50 MRSP isolates tested in this study exhibited a MIC for minocycline of 1.0 mg/L, which, according to the latest CLSI recommendations, should be reported as intermediate susceptibility. However, all three isolates carried two or three *tet* genes in different configurations, e.g., *tet*(K)-*tet*(M)-*tet*(O), *tet*(K)-*tet*(M), or *tet*(M)-*tet*(L). It should be emphasized that in each case, genetic determinants of minocycline resistance were present. These results suggest a possible revision of the minocycline breakpoints recommended by CLSI.

In one study conducted in Canada and the USA, resistance to minocycline was detected in 31% of MRSP isolates, which is a relatively low level of resistance for multidrug-resistant isolates. Similarly to our results, strains carrying the *tet*(M) gene were confirmed to be resistant to minocycline; the *tet*(K) gene was associated with susceptibility to this antimicrobial. However, detection of the *tet*(K) and *tet*(M) genes and only *dru* typing were used for molecular characterization. In some cases, the *tet*(M) gene was not detected in minocycline-resistant isolates, suggesting the presence of other *tet* genes [14]. It is also known that in different geographical regions, distinct sequence types with characteristic resistance profiles predominate in the MRSP population. This is confirmed by studies of MRSP isolates from Japan, in which phenotypic methods revealed minocycline resistance in 59% of strains [28]. The prevalence of minocycline resistance among MRSP isolates originating from different regions of the USA ranged from 73.6% to 78.6%, which is a higher level of resistance than described in this study [29]. A systematic review indicated that tetracycline resistance is more frequent in ST258 and ST45 (over 90%) compared to ST71 (51%), and that the *tet*(K) gene is associated with ST71, in contrast to *tet*(M), which is more common in other STs [2]. Although this review did not include minocycline, the occurrence of individual *tet* genes in different STs suggests a higher level of minocycline susceptibility in ST71 isolates.

Available literature indicates that substantial epidemiological shifts have occurred within the global population of clinical MRSP isolates, including changes in the predominant sequence types across different geographical regions. A recent meta-analysis demonstrated a significant worldwide increase in the incidence of ST551 after 2013 [30]. Isolates belonging to ST551 have been reported in Portugal, Sweden, and Poland [9,25,30]. The increase in ST551 prevalence has been particularly evident in Europe and has coincided with a decline in the abundance of ST71 [30]. Nevertheless, the factors underlying the epidemiological success of ST551 remain poorly understood. This phenomenon may, at least in part, be attributed to local selective pressure associated with antimicrobial use. ST551 strains most commonly carry the SCC*mec* type V cassette and frequently exhibit resistance to minocycline [30]. Although the present study is based on a relatively limited number of isolates, our findings are consistent with previously published data. Notably, the newly described ST2851 represents a single-locus variant of ST551 and belongs to clonal complex CC551. Similar changes in the MSRP population have been described in recent years in Sweden, where an increase in the frequency of ST551 occurrence has also been observed [9]. Kang et al. (2017) [31] reported that among MRSP strains in Korea, the most prevalent clonal complexes (CC) were CC568 and CC677, which differ significantly from CC occurring in Europe, North America and Asia. In this study, 86.6% of the MRSP isolates were tetracycline-resistant and only one isolate (1.6%) showed minocycline resistance. Moreover, SCC*mec* type V was the major cassette detected in 45% of the isolates [30]. These results confirm that the *tet* genes are not located within the SCC*mec* cassettes, but on mobile genetic elements. The *tet*(M) gene is often associated with Tn916, Tn5801, or 5801-like transposons. The *tet*(K) gene is located on small plasmids, such as those from the pSC family [31,32,33,34].

## 4. Materials and Methods

### 4.1. Bacterial Isolates

A total of 50 MRSP isolates were studied. The isolates were cultured between 2007 and 2024 from clinical materials taken from dogs by veterinarians and referred for microbiological examination at the Microbiological Diagnostic Laboratory, Faculty of Veterinary Medicine, Warsaw University of Life Sciences-SGGW. Each isolate originated from a different animal that belonged to different owners and had no apparent epidemiological relationship. All isolates were cultured on Columbia Agar supplemented with 5% sheep blood (Graso Biotech, Starogard Gdański, Poland), and incubated aerobically for 24 h at 35 °C ± 2 °C. Pure cultures were stored at −20 °C. Presumptive identification of *S. pseudintermedius* was based on standard bacteriologic methods (colony morphology on Columbia blood agar, Gram staining, catalase test) and confirmed by the PCR as described by Sasaki et al. (2010), involving species-specific amplification of thermonuclease gene fragment [35]. Methicillin resistance was determined by the disk diffusion method with oxacillin (1 µg, Oxoid, Basingstoke, UK), in accordance with the CLSI veterinary guidelines [26]. The presence of the *mecA* gene was tested using PCR method described by Strommenger et al. (2003) [36]. *S pseudintermedius* ATCC 49444 and *Staphylococcus aureus* ATCC 33592 were used as references in species-specific PCR and amplification of the *mecA* gene. Isolates obtained between 2007 and 2024 were selected for the study.

### 4.2. DNA Extraction from MRSP Isolates

Genomic DNA was extracted from all MRSP isolates with the Genomic DNA Mini Kit (A&A Biotechnology, Gdansk, Poland) using lysostaphin according to the manufacturer’s instructions, and the extracted DNA was stored at −20 °C.

### 4.3. Tetracycline Susceptibility Testing

Antimicrobial susceptibility testing (AST) for tetracycline (30 µg), doxycycline (30 µg), and minocycline (30 µg) was performed using the disk diffusion method [26]. All antimicrobial disks were purchased from Oxoid (Oxoid, Basingstoke, UK). Additionally, the minimum inhibitory concentrations (MICs) of tetracycline, minocycline, and doxycycline were evaluated using gradient strips (Liofilchem, Roseto degli Abruzzi, Italy). Briefly, suspensions of bacterial cells with a turbidity of a 0.5 McFarland standard were prepared and spread on Mueller-Hinton agar (MHA, Grasso Biotech, Poland). The disks and gradient strips were placed on inoculated MHA and subjected to aerobic incubation at 35 °C ± 2 °C. They were read after 20 to 24 h of incubation. Quality control was performed using the following reference strains: *S. aureus* ATCC 25923 for the disk diffusion method, *S. aureus* ATCC 29213 for MIC testing. Confidence intervals were calculated using the online sample size calculator tool (https://www.sample-size.net, accessed on 28 November 2025). The most current version of CLSI veterinary guidelines was used for the interpretation of the AST results (Table S2). Isolates in the intermediate category were classified as resistant for the purpose of analysis.

### 4.4. Detection of Tetracycline Resistance Genes

The presence of tetracycline resistance genes was determined by PCR amplification using the primers listed in Table 3.

### 4.5. Molecular Characterization of MRSP Isolates

Multilocus sequence typing of seven housekeeping genes (*tuf*, *cpn60*, *pta*, *purA*, *fdh*, *ack*, and *sar*) was used to determine the sequence types among examined MRSP [18]. The sequence types were assigned by comparing the obtained sequences with those available in the PubMLST database. Isolates with new combinations of alleles were submitted to the MLST database curator Vincent Perreten for assignment.

All the MRSP isolates were also subjected to SCC*mec* typing as previously described by Kondo et al. (2007) [20]. Additionally, SCC*mec* cassette type II–III was identified using the PCR method, as described by Descloux et al. (2008) [39].

## 5. Conclusions

In the case of multidrug-resistant MRSP isolates, each potential therapeutic option is particularly important; therefore, monitoring minocycline resistance and the prevalence of different *tet* genes among MRSP isolates is crucial. Therefore, for some strains of MRSP, minocycline may be a viable treatment option. Our results are in line with the literature data, which show a significantly higher incidence of minocycline susceptibility and tetracycline and doxycycline resistance in MRSP ST71-II–III. These isolates are typically characterized by the absence of the *tet* genes or the presence of a single *tet*(K) gene. Furthermore, isolates belonging to ST551 and ST2851, which carry SCCmec type V, had the *tet*(M) and *tet*(K) genes, or even three genes. Resistance to minocycline was found to be much more frequent in these isolates.

The results obtained in this study indicate that minocycline may have limited efficacy against MRSP infections in Poland. Nevertheless, in other geographic areas, specific genotypes—such as those carrying clonal complex 71 with the SCC*mec* type II–III cassette—may remain susceptible, making minocycline a potential therapeutic option. These findings underscore the importance of continuous surveillance of MRSP clones, including the distribution of *tet* genes and associated minocycline resistance profiles, to support evidence-based antimicrobial therapy. Furthermore, the results suggest that minocycline should be included in routine antimicrobial susceptibility testing of clinical MRSP isolates.

## Figures and Tables

**Table 1:** The minimal inhibitory concentration (MIC) ranges, MIC50, MIC90, and resistant isolates with confidence intervals among methicillin-resistant *Staphylococcus pseudintermedius* isolates (n = 50).

Antimicrobial	MIC Range (mg/L)	MIC_50_ (mg/L)	MIC_90_ (mg/L)	Resistant Isolates	Confidence Intervals CI 95%
Tetracycline	0.125–64	32	64	82%	0.71–0.93%
Doxycycline	0.032–16	8	16	82%	0.71–0.93%
Minocycline	0.016–6	6	12	56%	0.42–0.70%

**Table 2:** Molecular characterization of the clinical isolates of methicillin-resistant *Staphylococcus pseudintermedius* (n = 50).

Tetracycline Resistance Genes	Sequence Type	SCC*mec*	Number of Isolates
*tet*(K)	ST71	II–III	18
	ST71	IV	1
*tet*(K)-*tet*(M)	ST2851	V	8
	ST551	V	1
	ST71	II–III	1
*tet*(K)-*tet*(M)-*tet*(O)	ST551	V	7
	ST2851	V	1
*tet*(M)-*tet*(O)	ST551	V	1
	ST672	V	1
*tet*(M)-*tet*(L)	ST2869	V	1
*tet*(M)	ST258	IV	1
No tet genes detected	ST71	II–III	8
	ST71	IV	1

**Table 3:** Primers used for the detection of genes encoding resistance to antimicrobials from the tetracycline group among methicillin-resistant *Staphylococcus pseudintermedius* in this study.

Resistance Gene	Primer	Sequence (5′–3′)	Reference
*tet*(K)	tet(K)-1	TTAGGTGAAGGGTTAGGTCC	[37]
	tet(K)-2	GCAAACTCATTCCAGAAGCA	
*tet*(M)	tet(M)-1	GTTAAATAGTGTTCTTGGAG	[37]
	tet(M)-2	CTAAGATATGGCTCTAACAA	
*tet*(L)	Tet(L)-1	TCGTTAGCGTGCTGTCATTC	[38]
	Tet(L)-2	GTATCCCACCAATGTAGCCG	
*tet*(O)	Tet(O)-1	AACTTAGGCATTCTGGCTCAC	[38]
	Tet(O)-2	TCCCACTGTTCCATATCGTCA	

## Data Availability

Data is provided within the manuscript and supplementary information file.

## Supplementary Materials

The following supporting information can be downloaded at: https://www.mdpi.com/article/10.3390/antibiotics15010009/s1, Table S1: Row data. Table S2. The interpretative criteria used for susceptibility testing of methicillin-resistant *Staphylococcus pseudintermedius* isolates, based on CLSI VET01S [26].

## Abbreviations

The following abbreviations are used in this manuscript:

MRSP	Methicillin-resistant *Staphylococcus pseudintermedius*
MIC	Minimal inhibitory concentration
MLST	Multilocus sequence typing
SCC*mec*	Staphylococcal chromosomal cassette *mec*
ST	Sequence type
bp	Base pair
MIC_50_	Minimal inhibitory concentration at which 50% of a bacterial population is inhibited
MIC_90_	Minimal inhibitory concentration at which 90% of a bacterial population is inhibited
CI	Confidence interval
CLSI	Clinical and Laboratory Standards Institute
CC	Clonal complex
ATCC	American Type Culture Collection
AST	Antimicrobial susceptibility testing
MHA	Mueller–Hinton agar
