# Supplementary material for: Tetracycline Resistance Among Canine Methicillin-Resistant Staphylococcus pseudintermedius (MRSP) Clinical Isolates: Is Minocycline a Viable Treatment Option?
Source: Antibiotics (Basel). 2025 Dec 20;15(1):9. doi: 10.3390/antibiotics15010009 (PMC12838334; doi:10.3390/antibiotics15010009)
Supplement: Supplementary file 1 [file antibiotics-15-00009-s001.zip › Table S2.pdf]

Table S2. The interpretative criteria used for susceptibility testing of methicillin-resistant *Staphylococcus pseudintermedius* isolates, based on CLSI VET01S [26].

**Antimicrobial**	**Disk content (µg)**	**Interpretative criteria of inhibition zone diameter breakpoints (mm)**			**Interpretative criteria of MIC breakpoints (mg/L)**		
		**S**	**I**	**R**	**S**	**I**	**R**
Tetracycline	30	≥23	18-22	≤17	≤0.5	1-2	≥4
Doxycycline	30	≥25	21-24	≤20	≤0.12	0.25	≥0.5
Minocycline		NA	NA	NA	≤0.5	1	≥2

NA - Not Available
